# Correction: Brain interstitial pH changes in the subacute phase of hypoxic-ischemic encephalopathy in newborn pigs

**DOI:** 10.1371/journal.pone.0240643

**Published:** 2020-10-08

**Authors:** Gábor Remzső, János Németh, Viktória Varga, Viktória Kovács, Valéria Tóth-Szűki, Kai Kaila, Juha Voipio, Ferenc Domoki

In the Funding statement, the grant number from the funder EU-funded Hungarian grant is listed incorrectly. The correct grant number is: EFOP-3.6.1-16-2016-00008.

## References

[1] Remzső G, Németh J, Varga V, Kovács V, Tóth-Szűki V, Kaila K, et al (2020) Brain interstitial pH changes in the subacute phase of hypoxic-ischemic encephalopathy in newborn pigs. PLoS ONE 15(5): e0233851 10.1371/journal.pone.0233851 32470084 PMC7259698

